# Feasibility of a large cohort study in sub-Saharan Africa assessed through a four-country study

**DOI:** 10.3402/gha.v8.27422

**Published:** 2015-05-25

**Authors:** Shona Dalal, Michelle D. Holmes, Carien Laurence, Francis Bajunirwe, David Guwatudde, Marina Njelekela, Clement Adebamowo, Joan Nankya-Mutyoba, Faraja S. Chiwanga, Jimmy Volmink, Ikeoluwapo Ajayi, Robert Kalyesubula, Todd G. Reid, Douglas Dockery, David Hemenway, Hans-Olov Adami

**Affiliations:** 1 Department of Epidemiology, Harvard School of Public Health, Boston, MA, USA; 2 Channing Division of Network Medicine, Department of Medicine, Brigham and Women's Hospital and Harvard Medical School, Boston, MA, USA; 3 Centre for Evidence-Based Health Care, Faculty of Medicine and Health Sciences, Stellenbosch University, Cape Town, South Africa; 4 Department of Community Health, Mbarara University of Science and Technology, Mbarara, Uganda; 5 Department of Epidemiology & Biostatistics, Makerere School of Public Health, Kampala, Uganda; 6 Department of Physiology, Muhimbili University of Health and Allied Sciences, Dar es Salaam, Tanzania; 7 Institute of Human Virology, Abuja, Nigeria; 8 Greenebaum Cancer Center and Institute of Human Virology, University of Maryland School of Medicine, Baltimore, MD, USA; 9 Department of Internal Medicine, Muhimbili National Hospital, Dar es Salaam, Tanzania; 10 The South African Cochrane Centre, South African Medical Research Council, Cape Town, South Africa; 11 Department of Epidemiology and Medical Statistics, Faculty of Public Health, College of Medicine, University of Ibadan, Ibadan, Nigeria; 12 Department of Medicine, Makerere School of Medicine, Kampala, Uganda; 13 Department of Environmental Health, Harvard School of Public Health, Boston, MA, USA; 14 Department of Health Policy and Management, Harvard School of Public Health, Boston, MA, USA; 15 Department of Medical Epidemiology and Biostatistics, Karolinska Institutet, Stockholm, Sweden

**Keywords:** non-communicable, chronic disease, injury, South Africa, Nigeria, Tanzania, Uganda

## Abstract

**Background:**

Large prospective epidemiologic studies are vital in determining disease etiology and forming national health policy. Yet, such studies do not exist in sub-Saharan Africa (SSA) notwithstanding the growing burden of chronic diseases.

**Objective:**

We explored the feasibility of establishing a large-scale multicountry prospective study at five sites in four sub-Saharan countries.

**Design:**

Based on country-specific considerations of feasibility, Nigeria enrolled health care professionals, South Africa and Tanzania enrolled teachers, and Uganda enrolled village residents at one rural and one periurban site each. All sites used a 6-month follow-up period but different approaches for data collection, namely standardized questionnaires filled out by participants or face-to-face interviews.

**Results:**

We enrolled 1415 participants from five sites (range 200–489) with a median age of 41 years. Approximately half had access to clean-burning cooking fuel and 70% to piped drinking water, yet 92% had access to a mobile phone. The prevalence of chronic diseases was 49% among 45- to 54-year-olds and was dominated by hypertension (21.7% overall) – ranging from 4.5 to 31.2% across sites – and a serious injury in the past 12 months (12.4% overall). About 80% of participants indicated willingness to provide blood samples. At 6-month follow-up, 68% completed a questionnaire (45 to 96% across sites) with evidence that mobile phones were particularly useful.

**Conclusions:**

Our pilot study indicates that a large-scale prospective study in SSA is feasible, and the burden of chronic disease in SSA may already be substantial necessitating urgent etiologic research and primary prevention.

Non-communicable diseases (NCDs) already cause two-thirds of deaths worldwide (1). Projections indicate that by 2030 NCDs will be the cause of three of the four leading causes of death (2). Yet in sub-Saharan Africa (SSA) NCDs have been largely overlooked both in terms of local and international funding as well as research; the focus remains on communicable diseases and maternal and child health which predominate in statistics on premature mortality. However, data for NCDs, in particular, are sparse in SSA (3). The ongoing demographic transition in SSA has been a result of the reduction in communicable diseases, leading to an older population. At the same time, an epidemiologic transition from communicable to NCDs is occurring due to economic development and lifestyle changes (4).

The simultaneous communicable and NCD burden in SSA, and rapidly changing economic environment, have created a unique window of opportunity to study the epidemiologic transition with tools that were unavailable when current industrialized nations went through their transitions. Disparate lifestyles currently coexist within and between countries of the region – undernutrition with overweight, dietary changes and physical activity associated with subsistence farming and poverty, with those associated with sedentary lifestyles and wealth. The cohort design is ideal for the study of the epidemiologic transition, to identify etiology, and thus inform programs for the prevention of NCDs on this large, heterogeneous continent.

Large, long-term cohort studies in North America and Europe have been vital in determining disease etiologies, generating hypotheses for randomized controlled trials, and forming national health policy (5), yet cohorts of this scale (hundreds of thousands of people followed for 20 or more years) do not exist in SSA. Although future cohort studies have been proposed by public health experts (6, 7), few are planned in low- and middle-income countries. Apart from the prominent Birth-to-Twenty cohort study in South Africa which has successfully followed approximately 3,000 children from their birth in 1990 (8), research in SSA is dominated by cross-sectional surveys, case-control studies, and clinical trials (3), and has largely focused on communicable diseases.

We conducted a pilot study with the primary objective of determining the feasibility of enrolling and following persons for future long-term cohort studies on NCDs in Nigeria, South Africa, Tanzania, and Uganda, and to gather information on self-reported diagnoses of selected communicable and NCDs, their risk factors, injuries, mental health, and diet. Ensuring the successful follow-up of participants, crucial for any cohort study, is likely to be complex in SSA where traditional means of follow-up such as the mail may not be effective. Furthermore, in some low-income populations, there is significant migration. We therefore also intended to identify novel methods for the recruitment and retention of participants – particularly the use of mobile phones – and to determine willingness to participate in research with long-term follow-up.

## Methods

### Overview

We initiated pilot prospective cohorts of different populations at five sites in a partnership between the Harvard School of Public Health, USA; the Institute for Human Virology, Nigeria; Makerere University School of Public Health, Uganda; Mbarara University of Science and Technology, Uganda; Muhimbili University of Health and Allied Sciences, Tanzania; and Stellenbosch University, South Africa called the Africa/Harvard School of Public Health Partnership for Cohort Research and Training. Based on country-specific considerations for retention that is critical for a cohort study, populations were selected for educational background, diverse geographic and socioeconomic status, and established working relationships in the community. Nigeria enrolled health care professionals in two hospitals, South Africa enrolled teachers employed at public schools (primary, secondary, and intermediate), Tanzania enrolled primary school teachers, and Uganda enrolled village residents in periurban and rural locations. All sites used random selection for participants and a 6-month follow-up period. To accommodate local prerequisites, data collection approaches differed for each site as described in detail below.

Participant eligibility criteria were adults aged 18 years or older, and for the professional cohorts, were additionally those employed in schools (South Africa and Tanzania) or hospitals (Nigeria) at the time of data collection. Retired teachers and those due to retire in the following 6 months were excluded. Two sites in Uganda included current residents of villages who were not intending to relocate in the next 6 months. Informed consent was obtained from each subject either by voluntarily posting back a signed form with a completed questionnaire (South Africa and Tanzania), or through documentation with trained interviewers (Nigeria and Uganda).

### Questionnaire

We standardized an approximately 1-hour-long core questionnaire across all sites, with optional additional questions for individual sites. The questionnaire included sections on socioeconomic data, communicable and noncommunicable disease diagnoses, mental health and injuries, risk factors including smoking and alcohol use, and a comprehensive Food Frequency Questionnaire (FFQ). Some questions (e.g. on physical activity) were adapted from the World Health Organization STEPS instrument developed for use in resource-limited countries (9). The FFQ to collect information on dietary intake was specially designed and harmonized across all sites to allow cross-country comparisons.

English questionnaires were used in Nigeria and South Africa, and were translated and back translated into Kiswahili in Tanzania, Luganda and Runyakitara in Uganda, and into Afrikaans in South Africa. At enrollment, all participants were asked whether they were willing to complete a similar questionnaire after 6 months of follow-up.

In addition to the questionnaire, participants had their blood pressure, height, and weight measured by nurses or trained study staff following standardized procedures. Three sites (Stellenbosch University, Mbarara University of Science of Science and Technology, and Muhimbili University of Health Sciences) collected blood; Stellenbosch also collected urine samples to test for biomarkers and to establish willingness to provide biologic samples.

### Nigeria

Nigeria enrolled a cohort of nurses from two hospitals: one located in the capital city Abuja (National Hospital, Abuja), and the other, a semi-urban facility (University of Abuja Teaching Hospital, Gwagwalada) located 1.5 hours’ drive outside the city. Each hospital caters to an average population size of 2.2 million, with a combined total of 700 beds, and an average of 14,000 outpatients per month. Site investigators approached hospital administrators for approval to conduct the study and obtained a roster of professional staff. Two hundred randomly selected health professionals from the roster were then approached for participation at each hospital by trained study personnel. Eligible participating health professionals were interviewed face-to-face either at first contact or by appointment at a more convenient time. Biometrics (height, weight, and blood pressure) were measured after enrollment.

### South Africa

In South Africa, teachers from government schools in the urban Metro South District of the Cape Town Metropolitan area were invited to participate in the study. In 2008, there were approximately 25,500 state-paid teachers employed in the Western Cape Province, with almost two-thirds of these located at schools in four urban districts. The Western Cape Education Department approved and supported the study, and the relevant teacher trade unions were informed. We obtained a complete list of primary, intermediate, and secondary schools in the area, and selected all schools with 20 or more teachers for participation, resulting in 111 schools with approximately 3,166 teachers. The principals of the schools were invited through a letter describing the objectives and requirements of the cohort study and requesting written permission to enroll teachers from their school. Three rounds of invitations were sent during January–March 2011 and followed up by phone and email.

Questionnaire and informed consent packages were delivered to principals after confirming the number of teachers employed at each school. Principals were requested to distribute these packages to all the teachers. Willing teachers enrolled by completing the consent forms and a baseline questionnaire and returning these in a prepaid envelope to the study coordination center. During May–September 2011, appointments were made to visit schools at appropriate hours for trained nurses to collect physical measurements and biological samples from participants. Participants received the results of their blood pressure and laboratory results by personalized SMS or email according to their preference, and they were advised to see their regular physicians for readings that were outside the normal range for further management.

Teachers who indicated at baseline that they were willing to complete a follow-up questionnaire after 6 months received this by email or post according to their preference. Participants were reminded by phone, SMS, and email to return their documents. During March 2012, an information bulletin was emailed to participants and the director of the Metro South Education District to inform them of the progress in the study and to encourage follow-up participation.

### Tanzania

School teachers in Temeke District, Dar-es-Salaam, were first contacted for enrollment by one of two approaches: the District Education Office (DEO) or the teachers’ trade union (TTU). Of the 100 public schools in Temeke District, 18 were randomly selected to participate, and divided between union or DEO contact. The principals of selected schools received one packet containing a letter describing the study and sealed, prepaid envelopes for individual teachers from either the TTU or DEO. The individual teacher envelopes contained an informed consent form introducing the study, the baseline questionnaire, and an appointment card and letter to Temeke District Hospital to have their physical measurements and blood pressure measured. The study team met with the managers of the hospital to explain the study and requested that the staff assist participants in filling out the biological measurements form. Teachers were instructed to fill out the questionnaires on their own in private and to mail back completed informed consent forms and questionnaires in a stamped envelope. Teachers who were willing to be contacted again in 6 months were asked their preferred mode of contact (mobile phone, or email).

### Uganda

Ugandan participants were enrolled from two geographic areas, one in Wakiso District, a periurban community 30 min north of the capital Kampala, and the second in Bushenyi District, a rural southwestern district. The study team first met with community and district leaders and advertised the study widely to the community residing in the area. Villages in the district were selected by a multistage sampling approach.

In the periurban cohort of Wakiso District, two parishes consisting of 13 villages were randomly selected in Nangabo subcounty. Trained research assistants moved from house to house in these villages providing information about the study and explaining the objectives. Research assistants sought written consent from the head of household or the most responsible adult present to participate in the study. One consenting adult member was enrolled per household until the sample size of 300 was attained.

In the rural cohort of Bushenyi District, the study team enumerated the number of households in each village, and then randomly selected homes for approach. Trained interviewers went door to door to each selected home, and spoke with the head of household to describe the study. One consenting head of household was enrolled per home, with an effort made to balance the numbers of males and females. Those who agreed to participate signed an informed consent form; illiterate participants were read the informed consent form verbatim and signed with a mark.

In both cohorts, consenting household members completed a face-to-face interview. After the interview, the study interviewer measured height, weight, and blood pressure. In Bushenyi, willing participants also provided blood samples, which were transported the same day in a cold box to Mbarara University of Science and Technology for storage and analysis. All participants who agreed, were contacted a second time by direct face-to-face interview 6 months after their baseline assessment for a brief follow-up questionnaire addressing incident outcomes and diet.

This study was approved by the Harvard School of Public Health Institutional Review Board; the Institute of Human Virology Health Research Ethics Committee, Nigeria; the Health Research Ethics Committee of the Faculty of Health Sciences, Stellenbosch University; Makerere University School of Public Health Higher Degrees Research and Ethics Committee; National Institute for Medical Research, Tanzania; Mbarara University of Science and Technology Research Ethics Committee; and the Uganda National Council of Science and Technology.

We determined frequencies and their statistical significance using chi-square tests or the Fisher's exact test for small samples, and multivariate logistic regression to determine factors, their odds ratios, and 95% confidence intervals associated with participant follow-up. All data were analyzed using SAS (Cary, NC).

## Results

### Participant characteristics

In all, we enrolled 1415 participants through the different approaches, including 489 teachers in South Africa, 229 teachers in Tanzania, 200 nurses in Nigeria, 297 community members in periurban Uganda, and 200 in rural Uganda. At baseline, a total of 1229/1344 (91%) who answered the question, agreed to participate in a follow-up questionnaire after 6 months. The most frequently cited reasons for not agreeing to follow-up were that participants were too busy (52%) or that the questions were too personal (27%).

Participant characteristics, described in Table 1 included 14 ethnic groups. Approximately two-thirds were female, due to the predominance of females in the nursing and teaching professions. The median age was 41 years (IQR 32–49). Although the average number of living children per participant was 3, the range extended to 24 in the rural site; the age at first child's birth ranged from 12 to 42 years. Approximately half of all participants had access to clean-burning cooking fuel and 70% to piped drinking water, yet 92% had access to a mobile phone (either their own or through a family member or friend). The majority of respondents in all sites sought medical care at public facilities or clinics.

**Table 1:** Participant characteristics

	Rural Uganda	Periurban Uganda	Tanzania	Nigeria	South Africa	Total
	
Characteristic	*n* (%)	*n* (%)	*n* (%)	*n* (%)	*n* (%)	*n* (%)
Sex: female	100 (50)	158 (53)	191 (83)	133 (67)	344 (70)	926 (65)
Age, years						
18–24	29 (15)	58 (24)	0	6 (4)	8 (2)	101 (8)
25–34	59 (31)	83 (34)	57 (27)	67 (41)	35 (7)	301 (23)
35–44	62 (32)	47 (19)	75 (36)	55 (34)	167 (34)	406 (31)
45–54	32 (17)	28 (12)	63 (30)	34 (21)	205 (42)	362 (28)
55 +	9 (5)	27 (11)	14 (7)	2 (1)	71 (15)	123 (10)
Education						
No formal education/vocational/religious	33 (17)	22 (8)	1 (<1)	0	0	56 (4)
1–6 years	147 (74)	90 (31)	28 (13)	0	0	266 (20)
7–11 years	18 (9)	89 (31)	7 (3)	0	0	114 (9)
12+ (no college)	1 (<1)	66 (23)	180 (81)	2 (1)	92 (20)	341 (26)
College degree(s)	1 (<1)	11 (4)	7 (3)	159 (99)	337 (74)	515 (39)
Other	0	11 (4)	0	0	27 (6)	38 (3)
Marital status						
Never married	9 (5)	72 (24)	27 (12)	60 (32)	75 (16)	243 (18)
Married/living together	173 (87)	178 (61)	172 (76)	121 (64)	329 (69)	973 (70)
Separated, divorced, widowed, other	18 (9)	44 (15)	26 (12)	7 (4)	71 (15)	166 (12)
Number of persons in household						
1–4 persons	55 (28)	127 (44)	–	81 (47)	327 (69)	590 (52)
5–9 persons	124 (62)	137 (48)	–	79 (45)	144 (30)	484 (43)
10 +	20 (10)	24 (8)	–	14 (8)	5 (1)	63 (6)
Cooking fuel						
Gas/electric	0	8 (3)	–	114 (59)	473 (100)	595 (51)
Biogas	0	3 (1)	–	0	0	3 (<1)
Kerosene	0	4 (1)	–	75 (39)	0	79 (7)
Coal/charcoal	1 (<1)	204 (69)	–	5 (3)	0	210 (18)
Firewood/straw/dung	199 (100)	67 (23)	–	0	0	266 (23)
Other	0	10 (3)	–	0	1 (<1)	11 (1)
Drinking water						
Piped into dwelling/public tap/bottled	56 (28)	181 (61)	127 (56)	140 (76)	442 (98)	946 (70)
Well	67 (34)	97 (33)	79 (35)	39 (21)	0	282 (21)
Spring/surface/rainwater	36 (18)	18 (6)	20 (9)	3 (2)	0	77 (6)
Other	41 (21)	0	0	2 (1)	9 (2)	52 (4)
Access to cell phone^a^	173 (87)	258 (87)	–	174 (100)	436 (95)	1,041 (92)

a Participant's, family's, or friend's phone.

### Providing biological samples

Participants reported that they would be willing to provide samples of blood (993 of 1213 respondents; 82%), saliva (843/1142; 74%), urine (841/1126; 75%), nails (624/1028; 61%), and hair (557/984; 57%) in future research. During the study, 455 participants in South Africa (93%), 165 in Uganda (83%), and 165 (72%) in Tanzania provided blood samples.

### Follow-up

At 6-month follow-up, a total of 968 (68%) participants completed the questionnaire, and this varied considerably between sites. Tanzania and South Africa had similarly low follow-up at 114 participants (50%) and 221 (45%), compared to the other three sites: periurban Uganda had 253 (85%), rural Uganda had 188 (94%), and Nigeria had 192 (96%). In unadjusted analyses, there were significant differences in follow-up by sex, age, site, and chronic disease status, but in multivariable analyses the only factor remaining significant was site (Table 2). Although we could not assess the reasons for this difference directly because they are collinear, potential reasons are mode of contact (face-to-face vs. post) or the population followed (teachers vs. nurses and community residents).

**Table 2:** Multivariable logistic regression for factors associated with participant follow-up

Factor	Unadjusted OR	95% CI	Model 1^a^	95% CI	Model 2^b^	95% CI
Current age	0.97	0.96–0.98	1.01	0.99–1.03	1.02	1.00–1.04
Sex: female	0.76	0.59–0.96	1.18	0.88–1.59	1.05	0.73–1.53
Chronic disease	0.52	0.38–0.71			0.86	0.58–1.27
Site						
Tanzania	Ref.	–				
South Africa	0.83	0.61–1.14				
Periurban Uganda	5.85	3.87–8.83				
Rural Uganda	17.33	8.95–33.58				
Nigeria	24.21	11.4–51.44				

a Model 1: Covariates include age, sex, and site.

b Model 2: Covariates include age, sex, chronic disease, and site but does not include Nigeria as Nigeria did not include chronic disease questions.

OR, odds ratio; CI, confidence interval.

Contact through the mobile phone proved to be useful. Eighty percent of follow-up in Nigeria was done through phone calls due to nurse work schedules, whereas 20% were by face-to-face interviews. Furthermore, we found a proliferation and adoption of mobile phones: 45% of participants at our periurban Ugandan site and 53% at our rural site purchased a new mobile phone within the past 2 years, whereas 50% did in Tanzania, 69% in South Africa, and 93% in Nigeria. These numbers follow development trends of each country.

### Prevalence of self-reported health and disease

**Fig. 1:**
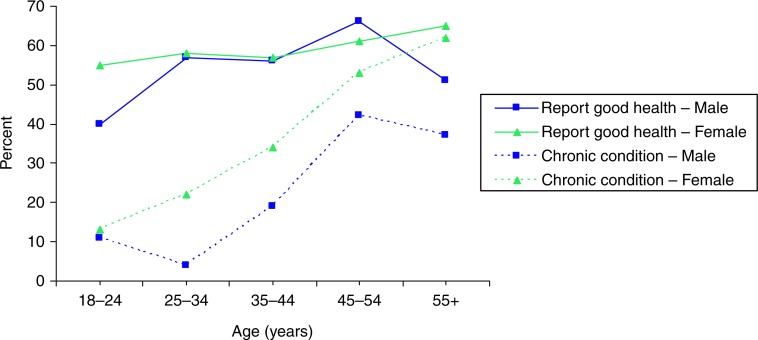
Self-reported health and chronic disease status by age and sex. Notes: Good health: Self-reported “good” or “very good” health on 5-point scale. Chronic disease: Self-reported diagnosis of hypertension, heart disease, stroke, diabetes mellitus, cancer, lung disease, or urinary incontinence.

**Fig. 2:**
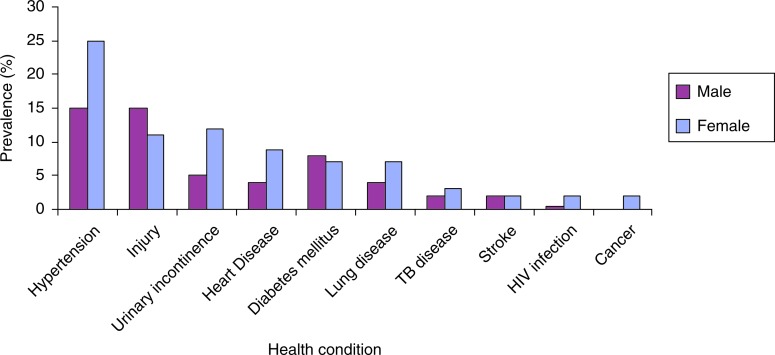
Self-reported diagnosis of communicable and non-communicable disease by sex.

In Nigeria, 90% of nurses rated their health ‘good’ or ‘very good’ compared to 74% of South African teachers, 47% of Tanzanian teachers, and only 33% of periurban and 38% of rural Ugandans, 18% of whom reported moderate or severe impairment. Although the prevalence of chronic diseases increased with age from 12% among 18- to 24-year-olds to 48% among those 55 years and older, the proportion of those reporting good health stayed fairly constant after age 25 (Fig. 1). South Africa consistently had the highest prevalence of NCDs (hypertension, diabetes mellitus, and cancer among the sites, Table 3). Tanzania had the next highest prevalence of most conditions, including a hypertension prevalence equivalent to South Africa (30% vs. 31%), and the highest prevalence of heart disease at 18.5%. Both Tanzanian and South African participants had a higher median age (40.5 and 46.5 years, respectively) than the other sites (rural Uganda: 36.6 years, periurban Uganda: 31.2 years, and Nigeria: 38 years). Rural Uganda had the highest prevalence of self-reported HIV infection; no participants reported HIV infection in South Africa, likely due to stigma. Women reported higher prevalence of most conditions than men, apart from diabetes mellitus and injuries (Fig. 2). We found 10% current smokers overall (range 1–19%) and 13% in rural Uganda where 79% reported growing up in a household with a smoker (results not shown).

**Table 3:** Self-reported diagnosis of communicable and non-communicable diseases by study site

	Rural Uganda	Periurban Uganda	Tanzania	Nigeria	South Africa	Total
	
Disease prevalence	% (*n*)	% (*n*)	% (*n*)	% (*n*)	% (*n*)	% (*n*)
HIV infection	4.5 (9)	<1 (1)	1.5 (3)	<1 (1)	0	1.1 (14)
TB disease	1.0 (2)	1.7 (5)	6.3 (13)	<1 (1)	2.7 (12)	2.5 (33)
Heart disease^a^	3.5 (7)	1.4 (4)	18.5 (38)	–	7.3 (29)	7.1 (78)
Hypertension	4.5 (9)	12.9 (38)	30.2 (65)	–	31.2 (140)	21.7 (252)
Diabetes mellitus	<1 (1)	3.0 (9)	7.6 (16)	–	13.5 (51)	7.1 (77)
Stroke	0	2.0 (6)	3.1 (7)	–	1.5 (5)	1.7 (18)
Cancer	<1 (1)	0	<1 (2)	–	2.5 (9)	1.1 (12)
Lung disease	1.5 (3)	<1 (1)	6.6 (15)	5 (10)	9.6 (43)	6.1 (72)
Injury	12 (24)	14.5 (43)	13.6 (25)	12.2 (22)	10.9 (48)	12.4 (162)
Urinary incontinence	7 (14)	1.3 (4)	12.3 (19)	4 (8)	14.7 (62)	9.7 (107)
Family history						
Heart disease^a^	21.5 (43)	12.0 (35)	26.4 (60)	5.7 (3)	42.1 (190)	27.0 (331)
Hypertension	25.0 (50)	47.3 (140)	40.5 (87)	45.5 (51)	63.4 (291)	48.3 (619)
Diabetes mellitus	9.5 (19)	22.7 (67)	25.2 (57)	22.5 (16)	52.6 (233)	31.7 (392)
Stroke	10.5 (21)	14.5 (43)	10.6 (24)	12.5 (7)	28.2 (126)	18 (221)
Cancer	3.5 (7)	7.7 (23)	11.9 (24)	6.3 (3)	29.1 (132)	15.8 (189)

a Includes angina, rheumatic heart disease, myocardial infarction, heart failure, or abnormal heart rhythm.

The prevalence of injuries that caused disruption to at least one day's activities was 12% overall, and similar across sites (Table 4). Overall, 22% of injuries resulted in disability and was highest in Tanzania at 52% (Table 4). On average, 8% of respondents had working firearms in their homes, only 12% wore helmets on motorbikes consistently, and 36% wore seatbelts consistently. The most frequent causes of the most serious injuries over the past 12 months were transport related (27%), sharp instruments (14%), and poisoning or drug overdoses (10%) (Table 5). Most (86%) injuries were accidents, 11% were self-inflicted, 4% were intentional, and most participants who experienced an injury sought medical care (81%).

**Table 4:** Self-reported injuries and safety practices by site

	Rural Uganda	Periurban Uganda	Tanzania	Nigeria	South Africa	Total
	
	% (*N*)	% (*N*)	% (*N*)	% (*N*)	% (*N*)	% (*N*)
Serious injury in past 12 months^a^	12 (24)	14 (43)	14 (25)	12 (22)	11 (48)	12 (162)
Disability from injury	17 (4)	33 (14)	52 (17)	13 (9)	18 (29)	22 (73)
Experienced domestic violence	8 (16)	5 (15)	–	5 (9)	3 (15)	5 (55)
Felt safe walking in neighborhood at night	54 (107)	45 (130)	77 (147)	51 (92)	31 (127)	48 (603)
Have working firearms in home	0	8 (23)	15 (31)	4 (6)	10 (45)	8 (105)
Wear helmet: always	1 (2)	8 (23)	6 (8)	14 (20)	52 (47)	12 (100)
Wear seatbelt: always	<1 (1)	8 (24)	13 (25)	80 (127)	65 (288)	36 (465)

a Injury which prevented normal activities for at least 1 day.

**Table 5:** Cause, type, and treatment of the most serious injury over past 12 months by sex

	Male	Female	Total	
		
Injury	% (*n*)	% (*n*)	% (*n*)	*p*
Serious injury in past 12 months^a^	15 (69)	11 (93)	12 (162)	0.06
Cause of most serious injury				0.11^b^
Transport related	34 (23)	22 (22)	27 (45)	
Sharp instrument (knife)	21 (14)	9 (9)	14 (23)	
Poisoning/drug overdose	6 (4)	13 (13)	10 (17)	
Burn	7 (5)	6 (6)	7 (11)	
Blunt instrument (club)	3 (2)	3 (3)	3 (5)	
Human fists/hands	6 (4)	4 (4)	5 (8)	
Animal bite	4 (3)	4 (4)	4 (7)	
Fall	4 (3)	4 (4)	4 (7)	
Firearm	1 (1)	4 (4)	3 (5)	
Other	13 (9)	30 (29)	23 (38)	
Injury type				0.35^b^
Accident	86 (59)	86 (83)	86 (142)	
Intentional	6 (4)	2 (2)	4 (6)	
Self-inflicted	9 (6)	12 (12)	11 (18)	
Injury treatment				0.17^b^
Sought medical help	78 (56)	83 (104)	81 (160)	
Traditional healer	10 (7)	6 (7)	7 (14)	
Home remedy	8 (6)	3 (4)	5 (10)	
No treatment sought	4 (3)	9 (11)	7 (14)	

a Injury which prevented normal activities for at least 1 day.

b Fisher's exact test.

## Discussion

Our pilot multicountry cohort study shows that enrolling and following professionals and geographic area residents in SSA for prospective research holds promise. Encouraging proportions of persons participated, provided biological samples, and were reached for follow-up. Using the mail in South Africa and Tanzania proved to be the most difficult of the contact approaches in obtaining timely responses. Multiple contacts and phone follow-up of participants, school principals, DEOs, and trade unions were required. Face-to-face follow-up was successful in Uganda as was a combination of mobile phone and face-to-face interviews in Nigeria. Mobile phones have growing influence in SSA (10) and could be a powerful tool for future research and public health intervention. As these regions experience more economic growth, we expect mobile phone technology to become even more pervasive, making it easier to establish dynamic and innovative longitudinal studies.

We found that successful follow-up differed significantly by mode of contact. Although participants showed willingness to participate in research, few returned questionnaires through the post or email. During sensitization meetings in Tanzania, teachers expressed discomfort in returning the questionnaire to the head teacher's office and preferred to use the post. However, the relative scarcity of post offices may have hampered the return of questionnaires. On the contrary, face-to-face interviews and mobile phones demonstrated high follow-up. Interestingly, sex, age, or having a diagnosed chronic condition was not associated with lower rates of follow-up after adjusting for site. Whether the reasons for the follow-up differences between sites can be predominantly attributed to the mode of contact is difficult to ascertain; other potential causes could be differences in population group (both South Africa and Tanzania enrolled teachers as compared to nurses and geographic residents in other sites), or other factors that were unique to those two countries. Further study is needed in these sites to determine the best means of follow-up for a full cohort study. Rapidly developing countries face the challenge of replicating longitudinal research in ways suitable for their context, given limited internet access and the obstacles to disseminating paper-based questionnaires. The collection of data through the cell phone, however, gives scientists the ability to ‘leapfrog’ over these obstacles and collect better and larger volumes of data to improve health. As smartphones become considerably cheaper and their use more widespread in resource-limited settings, these continuous behavior-sensing technologies will provide an enormous stream of data about human behavior (11). Sensors in phones enable the measurement of social activity and human physical activity, expanding our ability to understand a broader spectrum of risk factors and exposures for health outcomes (12). When these data are combined with genomics information and medical records of individuals, they have the potential to provide us with a far more complete picture of human health.

We found higher prevalence of non-communicable than communicable diseases among the adults in this pilot study. Due to a healthy worker effect, these estimates are likely lower in the professionally active cohorts than in the general population. However, they could be underestimated as NCDs are often detected later when complications are manifested. Nevertheless, South Africa, with older participants had the highest prevalence of NCDs, followed by Tanzania, then Nigeria, whilst village residents in Uganda had the lowest. In contrast to the sharp increase in the prevalence of chronic conditions by age, participant self-report of good health remained consistent. In previous studies, reporting of health status differs by country (13), education (14, 15), and sex (15). Our results indicate that older African individuals may be more tolerant of diseases associated with aging, and consider themselves in good health despite having these conditions.

Although we did not conduct diagnostic tests as part of our study, the self-reported prevalence of hypertension, diabetes, and stroke in our populations was similar to reports from other African studies (3), and in the case of hypertension (16), stroke (17), diabetes (18), and lung disease (19) were close to the rates seen in the United States. Screening programs in SSA have found high proportions of undiagnosed NCDs such as diabetes (20, 21) and hypertension (22), so the true prevalence may indeed be higher. The estimated cancer prevalence in our pilot study needs cautious interpretation because it is likely influenced by low awareness, stigma, and generally poor prognosis among cancer patients in these populations (23).

Although some risk factors for NCDs such as adiposity, physical inactivity, smoking, and alcohol use in Africa are likely similar to those seen in high-income countries, there are some key differences in the prevalence of these in Africa, and other risk factors that are unique to the African setting. For example, smoking is uncommonly reported in most countries other than South Africa (2). We found 10% current smokers (range 1–19%); interestingly the rural Ugandan site was unexpectedly high (13%), and had the highest reporting (79%) of growing up in a household with a smoker (results not shown). Daily physical activity although infrequently measured, is still common for the majority of the population as part of employment. Sub-Saharan African diets vary across the continent, are vastly different from Western diets, and may have different impacts on health. Furthermore, the changing work environments that come with economic growth will continue to change diets and physical activity as processed foods and sedentary lifestyles become more common. Finally, communicable diseases such as HIV/AIDS, TB, and malaria continue to be significant, and much about the interactions between these and NCDs remains to be explored. Identifying and determining the relative importance of these risk factors in SSA is imperative to guide policy makers in crafting prevention measures that could mitigate this burgeoning epidemic.

Our cohorts representing east, west, and southern Africa enable the study of groups at different stages of the epidemiologic transition, a rare opportunity in epidemiologic research. Establishing joint, large, longitudinal studies opens possibilities to examine both communicable and non-communicable diseases and their interactions, as well as in-depth examination of local diets, genetics, and environmental factors. The willingness of participants to provide biological samples across all sites is encouraging, and bodes well for future studies on biomarkers or gene-environment interactions. The widespread use of mobile phones allows for efficient collection of initial and follow-up survey information. Nested randomized intervention studies could further identify successful local prevention programs (24).

The study of injuries in SSA lags far behind the study of other health conditions. More than 12% of our study participants reported a serious injury in the past year, 22% of which resulted in a disability. Men had a higher prevalence of injuries than women. The most common cause of injuries in our study were transport related; globally there has been a 46% increase in deaths from road traffic accidents (3) and in Africa they cause the highest mortality (25). Road and traffic conditions are hazardous in many African countries, and laws are rarely policed. Country-specific studies have shown pedestrians and passengers of public transport are the most vulnerable (25). Yet, perceptions of risk are low, and few participants reported the use of safety measure such as helmets and seatbelts. Little is being done to address this enormous cause of early mortality in Africa.

Being a pilot study, our sample sizes at each site were small, limiting generalizability. However, the study was useful for purposes of testing methods for participant approach and participation, piloting questionnaires, providing an indication of disease prevalence, and determining the availability of mobile phone technology for data collection and thus future potential for launching cohort studies. Qualitative research may have provided more in-depth understanding on the reasons behind differential follow-up by method, but we did not conduct qualitative analyses in this study. The lack of reported HIV diagnoses in South Africa where HIV prevalence is high could have been due to stigma, or to the questions themselves. However, we were unable to assess the reasons for this and did not conduct HIV testing.

In conclusion, our multicountry pilot cohort study shows that both professionals and residents of geographic areas are willing to participate in longitudinal research and provide biological samples, and the majority can be reached and followed up by mobile phone. However, suboptimal follow-up in the teachers’ cohorts needs further examination. Our initial evidence suggests that NCDs, particularly hypertension and injuries, may already be common in SSA. The growing populations in the region are alone sufficient to stretch low resourced health care systems. The projected rapidly escalating NCD prevalence and continued burden of communicable diseases in SSA highlight the need for research to understand the etiology and regionally-important risk factors to prevent these diseases and health systems from being overwhelmed. The current environment in SSA offers a unique opportunity in history to study these conflating epidemics in the most diverse genetic population in the world, and may indeed provide clues to their control that can be applied to other world regions.

## References

[1] Lozano R, Naghavi M, Foreman K, Lim S, Shibuya K, Aboyans V (2012). Global and regional mortality from 235 causes of death for 20 age groups in 1990 and 2010: a systematic analysis for the Global Burden of Disease Study 2010. Lancet.

[2] World Health Organization Global burden of disease. Projections of mortality and causes of death, 2015 and 2030. http://www.who.int/healthinfo/global_burden_disease/projections/en/.

[3] Dalal S, Beunza JJ, Volmink J, Adebamowo C, Bajunirwe F, Njelekela M (2011). Non-communicable diseases in sub-Saharan Africa: what we know now. Int J Epidemiol.

[4] Adeyi O, Smith O, Robles S (2007). Public policy and the challenge of chronic noncommunicable diseases.

[5] Holmes MD, Dalal S, Volmink J, Adebamowo C, Njelekela M, Fawzi WW (2010). Non-communicable diseases in sub-saharan Africa: the case for cohort studies. PLoS Med.

[6] Collins FS (2004). The case for a US prospective cohort study of genes and environment. Nature.

[7] Potter JD (2004). Toward the last cohort. Cancer Epidemiol Biomarkers Prev.

[8] Richter L, Norris S, Pettifor J, Yach D, Cameron N (2007). Cohort profile: Mandela's children: the 1990 birth to twenty study in South Africa. Int J Epidemiol.

[9] World Health Organization STEPwise approach to surveillance. http://www.who.int/chp.steps/en.

[10] Idowu B, Adagunodo R, Adedoyin R (2006). Information technology infusion model for health sector in a developing country: Nigeria as a case. Technol Health Care.

[11] Pentland A, Lazer D, Brewer D, Heibeck T (2009). Using reality mining to improve public health and medicine. Stud Health Technol Inform.

[12] Madan A, Cebrian M, Moturu S, Farrahi K, Pentland S (2012). Sensing the “health state” of a community. Pervasive Computing.

[13] Jurges H (2007). True health vs response styles: exploring cross-country differences in self-reported health. Health Econ.

[14] Subramanian SV, Huijts T, Avendano M (2010). Self-reported health assessments in the 2002 World Health Survey: how do they correlate with education?. Bull World Health Organ.

[15] Banerjee D, Perry M, Tran D, Arafat R (2010). Self-reported health, functional status and chronic disease in community dwelling older adults: untangling the role of demographics. J Community Health.

[16] National Center for Health Statistics (2013). Health, United States, 2012: with special feature on emergency care. http://www.cdc.gov/diabetes/pubs/pdf/ndfs_2011.pdf.

[17] Fang J, Shaw KM, George MG (2012). Prevalence of stroke—United States, 2006–2010. Morb Mortal Wkly Rep.

[18] Centers for Disease Control and Prevention (2011). National diabetes fact sheet: national estimates and general information on diabetes and prediabetes in the United States.

[19] (2013). Estimated prevalence and incidence of lung disease. http://www.lung.org/finding-cures/our-research/trend-reports/estimated-prevalence.pdf.

[20] Mbanya JC, Motala AA, Sobngwi E, Assah FK, Enoru ST (2010). Diabetes in sub-Saharan Africa. Lancet.

[21] Motala AA, Esterhuizen T, Gouws E, Pirie FJ, Omar MA (2008). Diabetes and other disorders of glycemia in a rural South African community: prevalence and associated risk factors. Diabetes Care.

[22] Addo J, Smeeth L, Leon DA (2007). Hypertension in sub-saharan Africa: a systematic review. Hypertension.

[23] Knaul F, Adami HO, Adebamowo C, Arreola-Ornelas H, Berger A, Bhadelia A, FM Knaul, JR Gralow, R Atun, A Bhadelia (2012). Closing the cancer divide: an equity imperative. The global cancer divide: an equity imperative.

[24] Ioannidis JP, Adami HO (2008). Nested randomized trials in large cohorts and biobanks: studying the health effects of lifestyle factors. Epidemiology.

[25] Lagarde E (2007). Road traffic injury is an escalating burden in Africa and deserves proportionate research efforts. PLoS Med.

